# Supplementary material for: Tuning the gate and the gear: The LRRC26 (γ1) subunit modulates intrinsic gating and voltage-sensor coupling of the BK channel
Source: bioRxiv. 2025 Sep 6:2025.09.04.674290. Preprint. [Version 1] doi: 10.1101/2025.09.04.674290 (PMC12424821; doi:10.1101/2025.09.04.674290)
Supplement: Supplement 1 [file media-1.pdf]

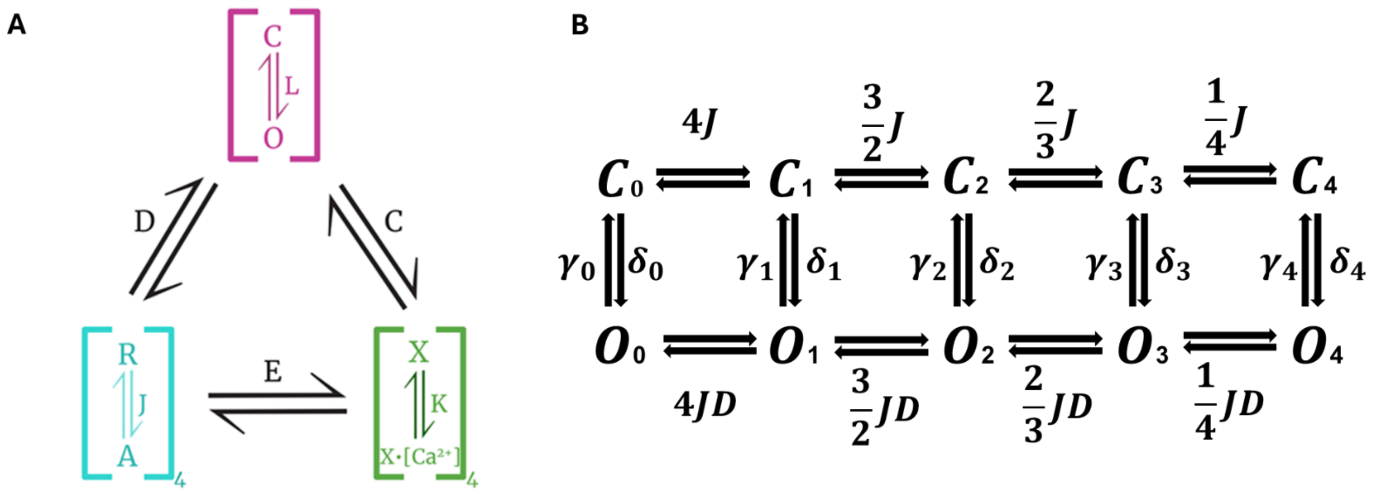

Supplementary Figure 1. HA allosteric gating model. (**A**) Scheme of HA allosteric model (*42*) indicates the possible conformations of the PD (C-O), VSD (R-A), and Ca^{2+} sensors (X-X·[Ca^{2+}]) in each of four α-subunits. The equilibrium constant, L, defines channel opening; The equilibrium constant J defines VSD activation; the binding constant K determines Ca^{2+} binding. The allosteric relationships between the modules are described by the allosteric factors D, C, and E. (**B**) Gating kinetics scheme. In this scheme, the horizontal C_i-C_i and O_i-O_i transitions are assumed to be in equilibrium for the C_i-O_i transitions, where δ_i and γ_i are the forward and backward rate constants defining the C_i–O_i transitions.

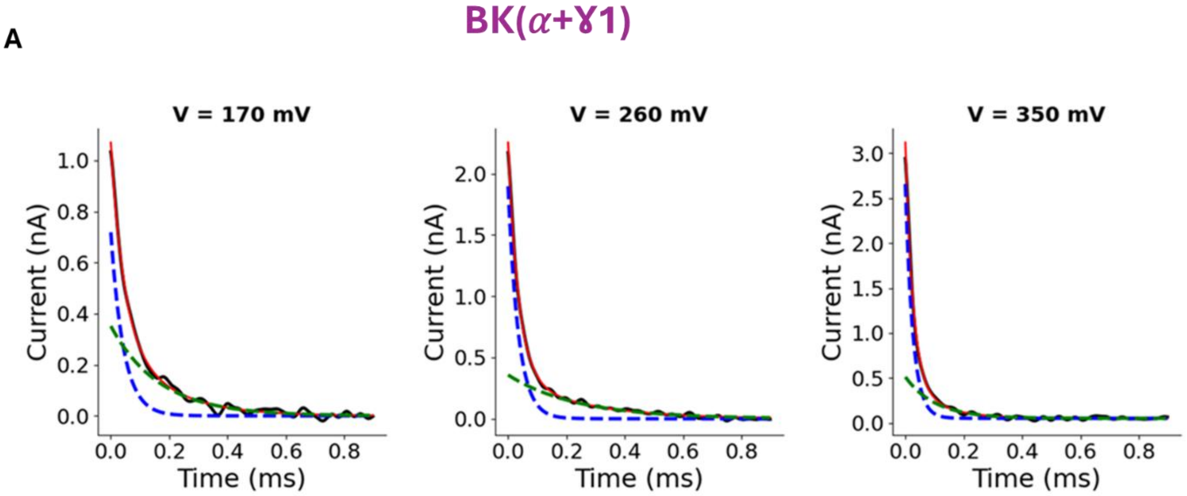

Supplementary Figure 2. Double exponential analysis of ON gating currents from BK(α+γ1) channels. (**A**) Representative biexponential fit (red) to gating currents (black) at three depolarizing pulses: 170 mV (left), 260 mV (center), and 350 mV (right). The function used for the biexponential fit was $I_g = A_{fast}e^{-t/\tau fast} + A_{slow}e^{-t/\tau slow}$ where A is the amplitude of each exponential. The gating current time constant (τ) for the fast (blue) and slow (green) component, respectively, resulted for each voltage was: 170 mV: τ-fast = 44 ± 2 µs; τ-slow = 167 ± 9 µs, 260 mV: τ-fast = 35.4 ± 0.4 µs; τ-slow = 237 ± 9 µs, and 350 mV: τ-fast = 25.5 ± 0.4 µs; τ-slow = 103 ± 6 µs.

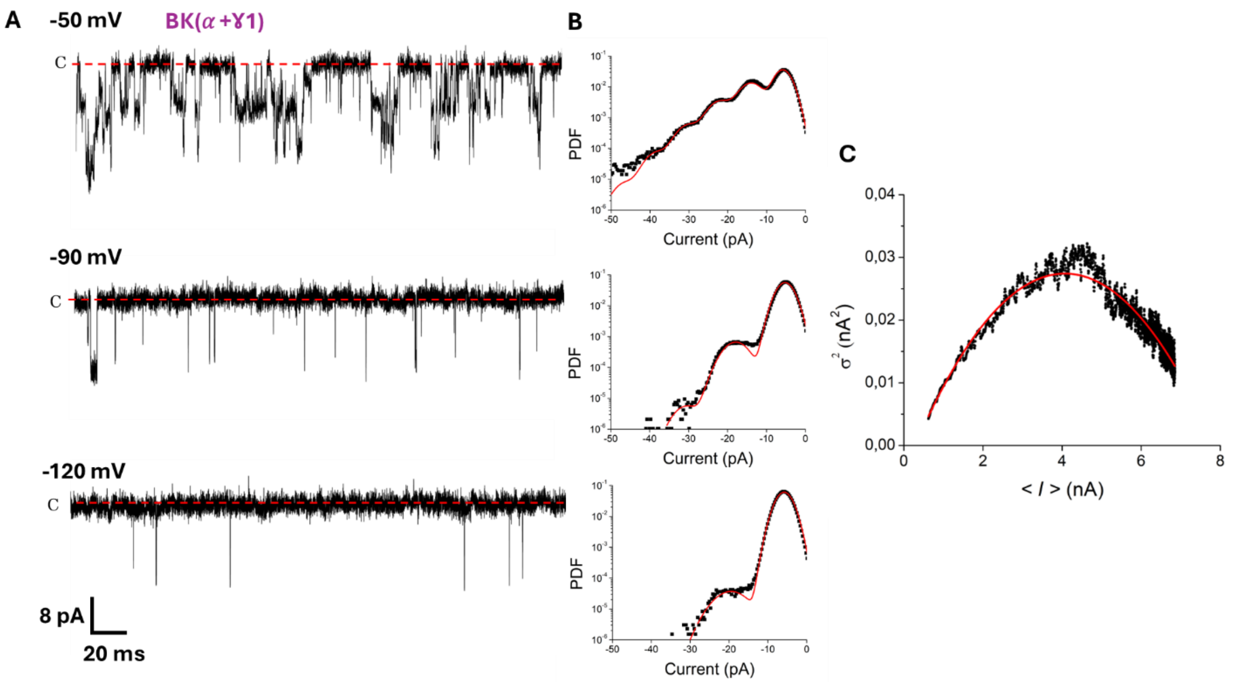

Supplementary Figure 3. Poisson distribution analysis for BK(α+γ1) unitary currents. (**A**) Unitary recordings for hundreds of channels of BK(α+γ1) were recorded in inside-out membrane patches at the indicated voltages and under symmetric 110 mM K^+ conditions and zero free internal Ca^{2+}. All-point histograms of the opening events were constructed using 10-20 s of the single-channel recordings on the left, as shown in (**B**), and fitted to a Poisson distribution to calculate NP_O at multiple voltages and construct P_O-V curves in the whole voltage range. **(C)** The number of channels (N) was obtained using non-stationary noise analysis on activation currents for each recording. In this example, the variance (σ^2)-mean current ($<I>$) data was fitted (red) using the relation: $\sigma^2 = i<I> - <I>^2/N$, where i is the single-channel current with i = 20 pA and N = 304 channels. $<I>$ was obtained from the average of 200 current records elicited using a 110-mV voltage pulse.

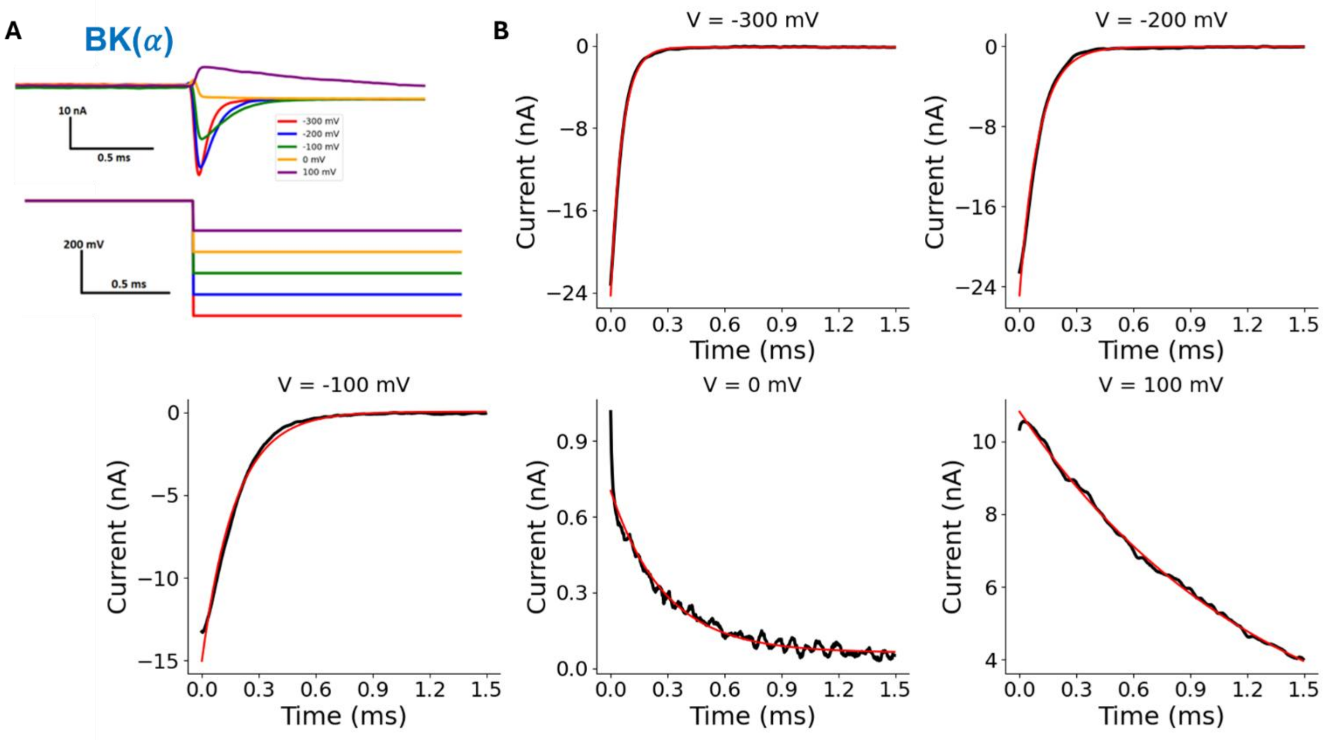

Supplementary Figure 4. Monoexponential analysis of deactivation kinetics from BK(α) channels. (**A**) Representative currents (top panel) of ionic currents from BK(α) channels, evoked in response to an instantaneous voltage protocol (bottom panel) at different hyperpolarization potentials (see the inset for color symbology). (**B**) Representative monoexponential fits (in red) to tail currents (in black) from (**A**). Time constants of deactivation were estimated for five different potentials: 59.3 ± 0.2 μs (-300 mV), 99.8 ± 0.7 μs (-200 mV), 175.6 ± 1.5 μs (-100 mV), 285± 6 μs (0 mV), and 1310 ± 20 μs (100 mV).

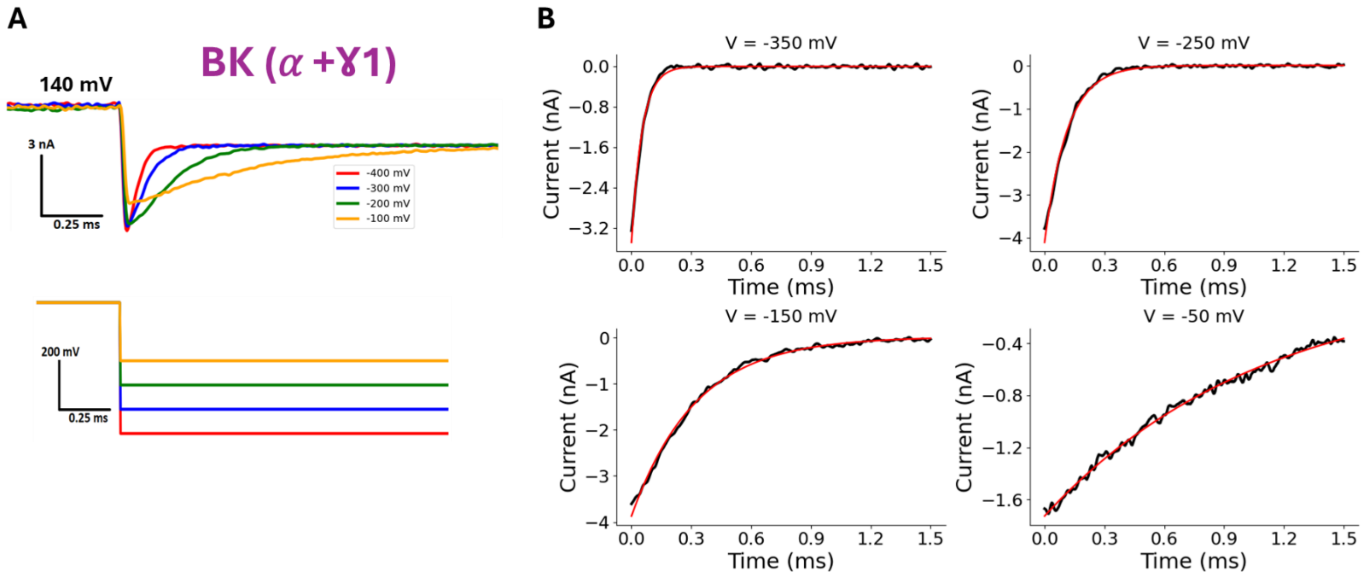

Supplementary Figure 5. Analysis of deactivation kinetics from BK(α+ γ1) channels. (**A**) Representative currents (top panel) of ionic currents from BK(α+γ1) channels, evoked in response to an instantaneous voltage protocol (bottom panel) at different hyperpolarization potentials (see inset for color symbology). (**B**) Representative monoexponential fits (in red) to tail currents (in black) from (**A**). Time constants of deactivation were estimated for five different potentials: 53.4 ± 0.4 µs (-350 mV), 113.3 ± 0.8 µs (-250 mV), 320.6 ± 2.3 µs (-150 mV), and 1091.2 ± 233 µs (-50 mV)

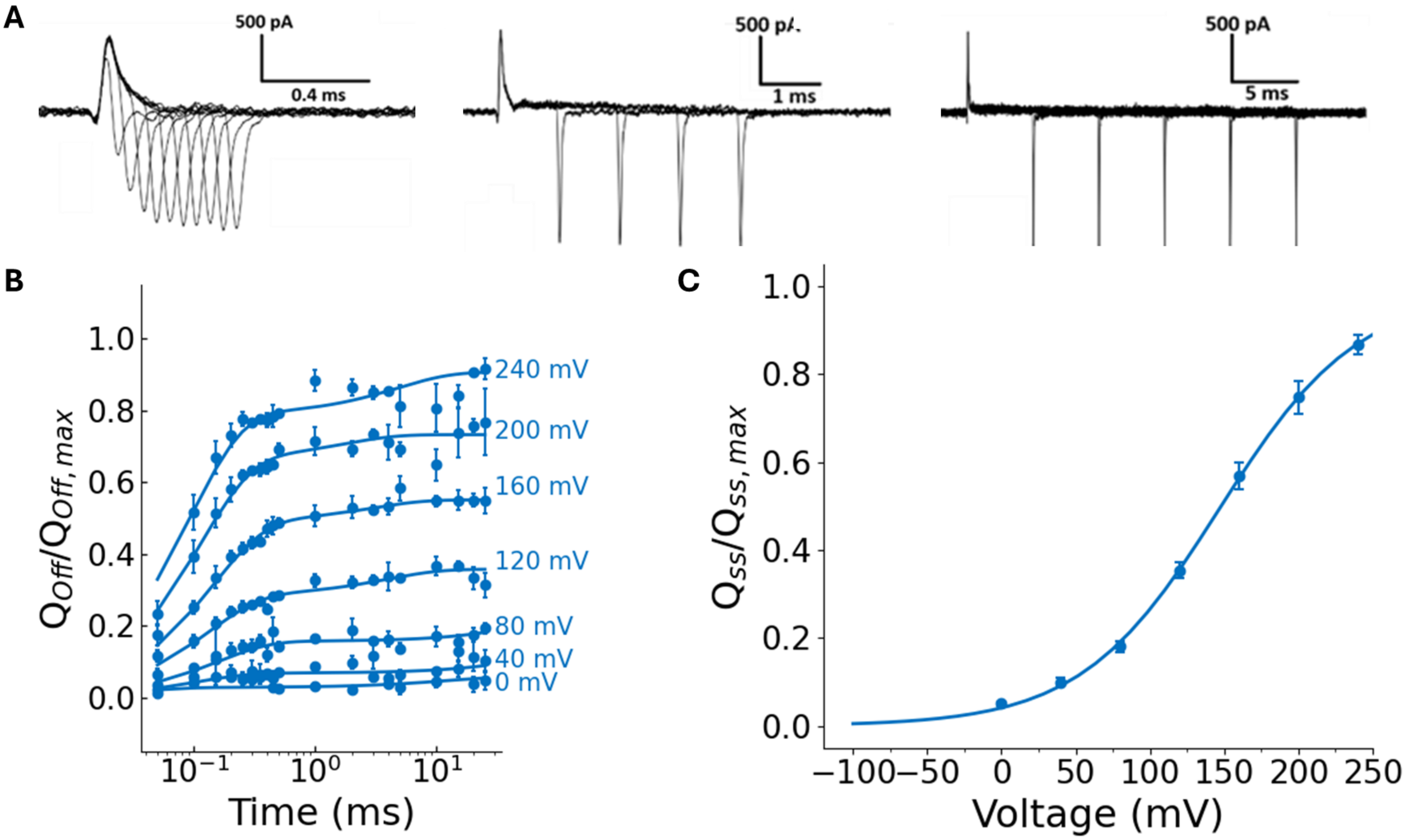

Supplementary Figure 6. BK(α) OFF-gating charge kinetics. (**A**) Representative records of gating currents of BK(α) channels, evoked at 200 mV, with different pulse length durations (from 0.05 ms to 25 ms). The total OFF-charge (Q_{off}) was determined by numerically integrating the OFF-gating current during 0.25 ms. (**B**) Q_{off} was normalized by $Q_{off,max}$ at 0, 40, 80, 120, 160, 200, and 240 mV, plotted against time (the length for each depolarization pulse; mean ± SEM, N = 4), and each dataset was fitted to a biexponential function (see **Methods**) for every voltage. (**C**) Normalized steady state OFF-charge (Q_{ss}) of BK(α) from experiments like in (**B**) (N = 4). Q_{ss} were fitted to a two-state model $Q_{ss}/Q_{ss,-max}$ = 1/[1+exp(-z_{Qss}F(V–V0.5)/RT)] with the following parameters: $V_{0.5}$ = 146.9 ± 2.8 mV and z_{Qss} = 0.544 ± 0.019.

Parameter	BK(α +ɣ1)	BK(α)
$V_{0.5}$ (mV)	80.9 ± 0.3	218.3 ± 0.8
z_G	1.37 ± 0.02	0.85 ± 0.02
$V_{H,}$ (mV)	185 ± 1.5	178 ± 0.4
z_Q	0.58 ± 0.04	0.59 ± 0.04
J_0	0.013 ± 0.001	0.029 ± 0.005
z_J	0.58 ± 0.04	0.59 ± 0.04
L_0	$4.5*10^{-4} \pm 2.9*10^{-4}$	$4.9*10^{-6} \pm 0.9*10^{-6}$
z_L	0.74 ± 0.06	0.36 ± 0.06
D	84.4 ± 6.4	25.6 ± 4.9

Supplementary Table 1. Summary of BK(α) and BK(α+γ1). $V_{0.5,}$ and V_H correspond to the half-activation voltages for G(V) and Q(V) curves (**Fig. 1**), respectively. The other parameters correspond to the HA model.

Parameter	BK(α +γ1)	BK(α)
$\gamma_{0,0}$ (s^{-1})	675.0 ± 41.1	3587.2 ± 68.8
z_{γ}	0.212 ± 0.005	0.136 ± 0.002
*$\delta_{0,0}$ (s^{-1})	0.3 ± 0.2	0.017 ± 0.004
*z_{δ}	-0.53 ± 0.06	-0.22 ± 0.06

Supplementary Table 2. Summary of BK(α+γ1) and BK(α) kinetic. Values (*) were calculated from $\gamma_{0,0}$ and L_0.

$[Ca^{2+}]_{Free}$ (μM)	BK(α +γ1) (mV)	BK(α) (mV)
0.01	80.9 ± 0.3 (9)	218.3 ± 0.8 (8)
0.1	55.9 ± 0.3 (8)	233.5 ± 8.5 (4)
0.5	12.7 ± 0.6 (5)	193.9 ± 1.7 (4)
0.8	-4.8 ± 0.8 (3)	
3	-37.8 ± 0.8 (5)	126.6 ± 1.3 (4)
10	-98.8 ± 1.5 (3)	
100	-154.7 ± 1.7 (7)	-35.6 ± 1.0 (4)
300	-165.2 ± 1.7 (4)	
1 (mM)	-197.7 ± 1.8 (7)	

Supplementary Table 3. Summary of BK(α+γ1) and BK(α) half activation voltages for a two-state model from the family of G(V)s at intracellular Ca^{2+} concentrations in the range of 0.01 μM -1 mM, followed by the number of replicas in parentheses.

Parameter	BK(α +ɣ1)	BK(α)
K_D (μM)	0.3 ± 0.2	1.0 ± 0.5
C	11.3 ± 1.1	11.5 ± 4.1
$*\,\Delta\Delta G(0.01\ - 100\, uM)$ (kcal/mol)	5.68 ± 0.04	5.9 ± 0.2

Supplementary Table 4. Summary of BK(α+γ1) and BK(α) Ca^{2+} dose-response parameters.
